# Trends in and determinants of visiting private health facilities for maternal and child health care in Nepal: comparison of three Nepal demographic health surveys, 2006, 2011, and 2016

**DOI:** 10.1186/s12884-020-03485-8

**Published:** 2021-01-03

**Authors:** Ramesh Prasad Adhikari, Manisha Laxmi Shrestha, Emily N. Satinsky, Nawaraj Upadhaya

**Affiliations:** 1 Suaahara II, Helen Keller International Nepal, Lalitpur, Nepal; 2 Padma Kanya Multiple Campus, Tribhuvan University, Kathmandu, Nepal; 3 Suaahara II, FHI360, Kathmandu, Nepal; 4 Center for Global Health, Massachusetts General Hospital, Boston, MA USA; 5 Department of Research and Development, HealthNet TPO, Amsterdam, the Netherlands

**Keywords:** Maternal and child health, Health-seeking, Private health care, Nepal, Public private partnership

## Abstract

**Background:**

Maternal and child health care services are available in both public and private facilities in Nepal. Studies have not yet looked at trends in maternal and child health service use over time in Nepal. This paper assesses trends in and determinants of visiting private health facilities for maternal and child health needs using nationally representative data from the last three successive Nepal Demographic Health Surveys (NDHS).

**Methods:**

Data from the NDHS conducted in 2006, 2011, and 2016 were used. Maternal and child health-seeking was established using data on place of antenatal care (ANC), place of delivery, and place of treatment for child diarrhoea and fever/cough. Logistic regression models were fitted to identify trends in and determinants of health-seeking at private facilities.

**Results:**

The results indicate an increase in the use of private facilities for maternal and child health care over time. Across the three survey waves, women from the highest wealth quintile had the highest odds of accessing ANC services at private health facilities (AOR = 3.0, 95% CI = 1.53, 5.91 in 2006; AOR = 5.6, 95% CI = 3.51, 8.81 in 2011; AOR = 6.0, 95% CI = 3.78, 9.52 in 2016). Women from the highest wealth quintile (AOR = 3.3, 95% CI = 1.54, 7.09 in 2006; AOR = 7.3, 95% CI = 3.91, 13.54 in 2011; AOR = 8.3, 95% CI = 3.97, 17.42 in 2016) and women with more years of schooling (AOR = 1.2, 95% CI = 1.17, 1.27 in 2006; AOR = 1.1, 95% CI = 1.04, 1.14 in 2011; AOR = 1.1, 95% CI = 1.07, 1.16 in 2016) were more likely to deliver in private health facilities. Likewise, children belonging to the highest wealth quintile (AOR = 8.0, 95% CI = 2.43, 26.54 in 2006; AOR = 6.4, 95% CI = 1.59, 25.85 in 2016) were more likely to receive diarrhoea treatment in private health facilities.

**Conclusions:**

Women are increasingly visiting private health facilities for maternal and child health care in Nepal. Household wealth quintile and more years of schooling were the major determinants for selecting private health facilities for these services. These trends indicate the importance of collaboration between private and public health facilities in Nepal to foster a public private partnership approach in the Nepalese health care sector.

## Background

Health-seeking behaviour has been defined as a series of actions undertaken to remedy perceived health problems [1]. Several proposed conceptual models illustrate the determinants of health care utilisation; of these, the behavioural model [2], the health belief model [3], and the economic model [4] are widely accepted. Andersen’s Behavioural Model envisages three major components of health care utilisation: predisposing factors (e.g. age, sex, family size, education, employment), enabling factors (e.g. income, insurance, residence), and need factors (e.g. perceived health status, symptoms, days disabled due to illness). These factors combine to determine the use or non-use of health care services [2], and this model has been used extensively in studies investigating health service utilisation [5]. The health belief model centers individual perception, which is largely influenced by the individual’s perception of a particular health threat and the need to take appropriate action. This model has primarily been used to examine the utilisation of preventive care [3]. In contrast, the economic model assumes that economic determinants, including price, income, and other sociodemographic factors determine health care service utilisation [4, 6].

Health care service utilisation depends on physical, socio-economic, demographic, cultural, and political factors. These factors, including cultural beliefs and practices around health care utilisation, are closely associated with and determined by the health care system in specific countries [7–9]. In many low- and middle-income countries (LMICs), illiteracy, poverty, and under-funding of the health sector influence health-seeking behaviours [10–13]. Additionally, cost, service quality, service providers’ behaviours, patient education level, knowledge of illness and wellbeing, and cultural beliefs influence where an individual seeks health services [14, 15].

Maternal health services, including antenatal care (ANC), delivery, and postnatal care (PNC), as well as child health services such as immunisation and treatment for diarrhoea, pneumonia, and malaria, are available at both public and private facilities. Providers at private facilities deliver a significant portion of health care services in LMICs, including in both rural and urban areas and for different ethnicities and socioeconomic groups [16]. Research on the equity of private health care, however, is inconclusive, with wide variation in provision of services across settings [17]. While some analyses have shown that provision of care in the private sector is inherently inequitable, with wealthy individuals accessing more services [18], other studies have shown that the private sector can improve equity in health services [19, 20]. For instance, the private sector may be better positioned than the public sector to deliver certain services and also has the flexibility to contribute to health financing [21]. However, this largely depends upon national governments enacting proper regulatory mechanisms for private health facilities to maximize the availability of these services in remote areas without compromising service quality.

Quality of care, and in particular, perceived quality based on patient evaluations, is an important deciding factor in choosing health care services [22]. The quality of services offered at health facilities impacts the demand for and use of these services, even in LMICs [23]. Globally, over half of all care for children with diarrhoea, fever, and cough is provided in the private sector, with more people from urban and wealthier backgrounds using these services than people from rural and poorer backgrounds [24]. While governments and policymakers invest more in public sector service delivery, there is growing interest in how the private sector can complement the public sector in health care delivery [25]. Throughout Nepal, guaranteeing access to high-quality health care has been a continual struggle in the public sector. Therefore, Nepal has developed a health sector strategy that states the importance of aligning the private sector with public services along four specific areas of operation: sustainable financing, integrated health care delivery, quality assurance, and technological innovation [26].

In Nepal, there are three primary types of health facilities: 1) government owned public health facilities; 2) privately owned health clinics and hospitals; and 3) faith-based and NGO operated health facilities. Kariki and Kadariya described the private health sector in Nepal as encompassing formal hospitals, nursing homes, private practitioners and pharmacists, private medical colleges, NGO- or community-run hospitals, and informal practitioners (e.g., faith healers) [27]. For this paper, the private sector includes any privately owned health facility where patients are expected to cover all health care bills themselves.

Prior to 1991, the health system in Nepal was primarily government owned, with only 16 private hospitals nationwide in 1990. The National Health Policy in 1991 paved the way for increased investment in the private sector [28]. This resulted in a rapid increase in private hospitals, with 301 established by 2014. Currently, over two thirds of hospital beds are in private health facilities, and 60% of Nepal’s doctors work in the private sector [29]. Financing mechanisms for health services include government subsidies for essential health care (provided free of charge from government health facilities) and out-of-pocket payment by the patient and/or their family members. Health care bills for all services in private health facilities and some specialized services in government owned public health facilities are covered by the patient and/or their family members. In February of 2015, the government rolled out the Social Health Security scheme for services that are beyond the essential health care package. This was intended to increase financial protection by promoting pre-payment and risk pooling in the health sector [30]. However, this scheme is only applicable in government designated health facilities. There are no financial risk protection mechanisms for health care bills paid at private health facilities.

In Nepal, available data indicate that the number and share of private services in health provision has increased in recent decades, particularly for ambulatory care. Of the total health expenditure in Nepal, private facilities share 70%, of which about 85% comes from out-of-pocket payments; this indicates a shift in the role of private facilities in health provision in Nepal [27]. However, no research has yet estimated trends in and determinants of utilisation of private health facilities using nationally representative survey data in Nepal. Hence, our study aimed to explore trends in and determinants of maternal and child health service utilisation from private health facilities using the last three nationally representative Nepal Demographic Health Surveys conducted in 2006, 2011, and 2016.

## Methods

This paper analysed data from the Nepal Demographic Health Survey (NDHS) conducted in 2006, 2011, and 2016. The NDHS is a nationally representative cross-sectional household survey which collects information on health and socio-demographic information at the national and sub-national levels. The NDHS applies a two-stage cluster sampling technique. The enumeration areas are selected in the first stage based on probability proportion to size (PPS). Households are then selected from each cluster based on equal probability [31].

Of all women interviewed with the NDHS, 4066 women provided information on ANC services in 2006, 4148 women in 2011, and 3998 women in 2016 (Table 1). Likewise, information related to place of delivery was collected from 5545 women in 2006, 5391 women in 2011, and 5060 women in 2016. Information on services for child sickness was collected from 5252 women in 2006, 4040 women in 2011, and 4887 women in 2016. Each NDHS collected data on ANC service use and delivery care for female participants’ most recent birth in the 5 years preceding the survey. Data on treatment utilisation for child illnesses were collected for all children under 5 years of age, living in each household, who received care in the 2 weeks prior to the survey.

**Table 1:** Sample size distribution by NDHS survey year

Year	Women interviewed	Information on ANC services	Information on delivery services	Information on child sickness services
**2006**	10,793	4066	5545	5252
**2011**	12,674	4148	5391	5140
**2016**	12,862	3998	5060	4887

Maternal and child health-seeking behaviours, the primary outcome variables, were measured using data on place of ANC services, place of delivery, and place of treatment for child diarrhoea and fever/cough. Place of health-seeking was categorized into two groups: 1) public (government hospitals, primary health care centers, health post, sub-health post, primary health care outreach clinics); and 2) private (private hospitals, nursing homes, polyclinic, non-governmental organization-run health facilities, private pharmacies).

Potential socio-economic confounders were selected based on prior knowledge of the socio-demographic and economic context. The major socio-economic confounders selected included wealth quintile (poorest, second poorest, middle, second richest, richest); caste/ethnicity (Dalit, Janajati, Brahman/Chhetri, other); mother’s completed years of schooling; mother’s age; headship of the household (male or female); urban versus rural residence; and agroecological zone (mountain, hill, terai).

The data analysis plan was designed based on the Zweifel economic model. The model indicates that economic, demographic, and social factors combine to determine health-seeking practices [2, 4]. We examined trends in health-seeking from private health facilities over time. Bivariate and multivariate logistic regression models were fitted to illustrate trends in and possible determinants of maternal and child health-seeking behaviours from private health facilities. A binary variable was created, with 1 specifying health-seeking from private health facilities and 0 specifying health-seeking from government institutions, or other. In the final analyses, women who did not receive ANC services and whose children did not receive care for diarrhoea and fever/cough were excluded. The weight sample was used for the analyses and the final models were adjusted for survey design effect. Independent variables in the final model were selected after checking for collinearity. Data analyses were performed in Stata version 14 (College Park, Texas).

## Results

**Fig. 1:**
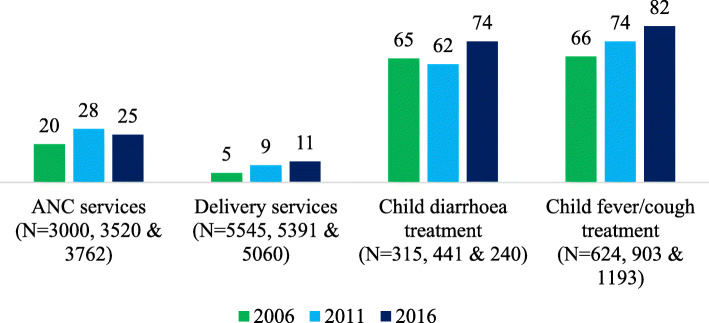
Percent of women seeking ANC services, delivery services, treatment for child diarrhoea, and treatment for child fever/cough at private facilities across survey years

Among all women who provided data on ANC service use (including those who did not receive ANC services; *N* = 4066), the percentage of women who received ANC services from private facilities increased from 14.7% in 2006 to 23.8% in 2016. When restricted to women who received ANC services (*N* = 3000), the percentage of women who received services from private facilities increased from around 20% in 2006 to 25% in 2016. Similarly, delivery in private facilities increased from 4.6% in 2006 to 10.8% in 2016 (Table 2, Fig. 1).

**Table 2:** Facility accessed for ANC, delivery, and child treatment

	2006	2011	2016
	N (%)	N (%)	N (%)
**Place of ANC services**			
None received	1066 (26.2)	628 (15.2)	236 (5.9)
Government institution	2297 (56.5)	2474 (59.6)	2689 (67.3)
Private institution	598 (14.7)	996 (24.0)	952 (23.8)
Other	105 (2.6)	50 (1.2)	121 (3.0)
Total	4066 (100.0)	4148 (100.0)	3998 (100.0)
**Place of delivery**			
Government institution	724 (13.1)	1399 (26.0)	2183 (43.1)
Private institution	256 (4.60)	506 (9.40)	549 (10.9)
Other	4565 (82.3)	3486 (64.6)	2328 (46.0)
Total	5545 (100.0)	5391 (100.0)	5060 (100.0)
**Child illness**			
Suffered from diarrhoea	623 (11.9)	711 (13.8)	371 (7.6)
Suffered from fever/cough	1212 (23.1)	1442 (28.1)	1420 (29.1)
**Total**	5252 (100.0)	5140 (100.0)	4887 (100.0)
**Place of diarrhoea treatment**			
None received	308 (49.4)	270 (38.0)	131 (35.2)
Government institution	112 (18.0)	158 (22.1)	48 (12.9)
Private institution	203 (32.7)	272 (38.3)	177 (47.8)
Other	–	11 (1.6)	15 (4.10)
**Total**	623 (100.0)	711 (100.0)	371 (100.0)
**Place of fever and cough treatment**			
None received	588 (48.5)	539 (37.4)	227 (16.0)
Government institution	206 (17.0)	206 (14.3)	196 (13.8)
Private institution	412 (34.0)	674 (46.7)	978 (68.8)
Other	6 (0.50)	23 (1.6)	20 (1.4)
Total	1212 (100.0)	1442 (100.0)	1420 (100.0)

Information on place of treatment for diarrhoea and fever/cough were collected from mothers who had a child under 5 years of age who experienced symptoms in the 2 weeks prior to completing the survey (again, including children who did not receive care for these illnesses). Among all children who suffered from diarrhoea, utilisation of private facilities for treatment increased from 32.7% in 2006 to 47.8% in 2016. Likewise, among children who suffered from fever/cough, utilisation of private facilities for treatment increased from 34.0% in 2006 to 68.8% in 2016. When restricted to children who actually sought treatment for these illnesses, the percent of children receiving services from private facilities for child diarrhoea and fever/cough both increased over this time period (diarrhoea, 65% in 2006 to 74% in 2016; fever/cough, 66% in 2006 to 82% in 2016) (Table 2, Fig. 1).

Disaggregated results indicate that the percent of women receiving services from private facilities increased among middle wealth quintile households (12.5%), mothers age 25 and above (8.5%), male headed households (6.8%), and terai agroecological zones (11.1%) between 2006 and 2016. Likewise, seeking delivery services from private facilities increased among middle wealth quintile households (10.3%), Janajati caste/ethnic groups (7.9%), mothers age 30 and above (8.7%), male headed households (7.4%), and terai agroecological zones (7.0%) during the same period. Seeking child diarrhoea treatment at private facilities increased among the second poorest wealth quintile households (16.1%), Dalit caste/ethnic groups (10.9%), mothers with 1–5 years of schooling (19.2%), mothers younger than 20 (34.5%), male headed households (12.3%), and terai agroecological zones (9.0%); similarly, seeking child fever/cough treatment at private facilities increased sharply among middle wealth quintile households (21.6%), mothers with no schooling (19.5%), mothers age 30 and above (18.3%), male headed households (17.8%), rural residence (19.2%), and mountain agroecological zones (29.4%) from 2006 to 2016 (see online supplementary material, Tables 1–4).

### Determinants of health-seeking from private health facilities

The adjusted logistic regression models show that in all three surveys, higher household wealth quintile was statistically significantly associated with utilisation of private facilities for ANC services (Table 3). Compared with women from the poorest households, women from the richest households were three times more likely to seek ANC care at private facilities in 2006 and six times more likely in 2011 and 2016 (Table 3).

**Table 3:** Associations between seeking ANC services from private facilities and household sociodemographic characteristics

	2006 (***N*** = 3000)	2011 (***N*** = 3520)	2016 (***N*** = 3762)
Household characteristics	AOR (95% CI)	AOR (95% CI)	AOR (95% CI)
**Wealth quintile**			
Poorest (*reference*)			
Second poorest	1.13 (0.68–1.87)	1.14 (0.78–1.67)	2.23** (1.50–3.31)
Middle	1.40 (0.87–2.25)	1.79** (1.21–2.65)	3.00** (2.03–4.43)
Second richest	2.14** (1.34–3.42)	3.14** (2.08–4.72)	3.73** (2.47–5.63)
Richest	3.01** (1.53–5.91)	5.56** (3.51–8.81)	6.00** (3.78–9.52)
**Caste/ethnicity**			
Dalit *(reference)*			
Janajati	0.92 (0.61–1.39)	0.77 (0.54–1.10)	1.38 (0.92–2.07)
Brahmin/Chhetri	0.95 (0.62–1.47)	1.05 (0.74–1.48)	1.61* (1.09–2.36)
Other	0.83 (0.50–1.40)	1.24 (0.77–1.99)	2.37** (1.58–3.56)
**Mother’s years of schooling**	1.15 (1.10–1.19)	1.06** (1.02–1.09)	1.05 (1.02–1.08)
**Mother’s age**	0.99 (0. 96–1.01)	1.00 (0.98–1.03)	1.00 (0.98–1.02)
**Female household head**	1.19 (0.89–1.58)	01.13 (0.90–1.42)	0.95 (0.77–1.16)
Rural as the place of residence	0.92 (0.54–1.55)	0.97 (0.73–1.29)	0.98 (0.71–1.35)
**Agroecological zone**			
Mountain (*reference*)			
Hill	1.33 (0.57–3.11)	1.52 [0.91–2.55]	3.25** (1.58–6.69)
Terai	1.68 (0.72–3.92)	2.33** [1.38–3.92]	4.11** (1.98–8.54)

* *p* < 0.05; ** *p* < 0.01

In the adjusted logistic regression models, women from the richest households were more than seven times as likely to have delivered their child at a private facility in 2011 and 2016 compared with women from the poorest households (Table 4). In 2016, women from the richest households were eight times as likely as women from the poorest households to access these services. Additionally, the more years of schooling women had received was also statistically significantly associated with delivering at a private facility in all three survey waves.

**Table 4:** Associations between delivering a child at private facilities and household sociodemographic characteristics

	2006 (***N*** = 5545)	2011 (***N*** = 5391)	2016(***N*** = 5060)
**Household characteristics**	**AOR (95% CI)**	**AOR (95% CI)**	**AOR (95% CI)**
**Wealth quintile**			
Poorest (*reference*)			
Second poorest	1.39 (0.60–2.94)	1.40 (0.78–2.49)	2.94** (1.52–5.69)
Middle	1.23 (0.56–2.72)	2.28* (1.28–4.07)	7.36** (3.74–14.47)
Second richest	2.17* (1.08–4.37)	4.28** (2.32–7.88)	8.16** (4.22–15.79)
Richest	3.31** (1.54–7.09)	7.27** (3.91–13.54)	8.34** (3.97–17.42)
**Caste/ethnicity**			
Dalit (*reference*)			
Janajati	1.58 (0.71–3.50)	1.16 (0.72–1.86)	1.91* (1.16–3.15)
Brahmin/Chhetri	1.91 (0.98–3.72)	1.33 (0.78–2.26)	1.69* (1.01–2.82)
Other	1.68 (0.65–4.33)	1.09 (0.54–2.23)	1.48 (0.86–2.57)
**Mother’s years of schooling**	1.22** (1.17–1.27)	1.09** (1.04–1.14)	1.11** (1.07–1.16)
**Mother’s age**	1.00 (0.97–1.04)	1.01 (0.98–1.04)	1.03* (1.00–1.06)
**Female household head**	1.88* (1.19–2.97)	1.14 (0.79–1.63)	0.78 (0.57–1.05)
**Rural residence**	1.12 (0.65–1.93)	0.81 (0.56–1.18)	1.19 (0.82–1.72)
**Agroecological zone**			
Mountain (*reference*)			
Hill	1.06 (0.42–2.68)	1.11 (0.64–1.93)	3.46** (1.59–7.52)
Terai	2.14 (0.85–5.35)	2.49** (1.42–4.35)	4.20** (1.88–9.38)

* *p* < 0.05; ** *p* < 0.01

No sociodemographic characteristic consistently predicted receiving treatment for child diarrhoea at a private health facility across all three survey waves. However, in 2006 and 2016, children from the richest households were respectively eight and six times more likely than children from the poorest households to receive treatment for diarrhoea at private facilities (Table 5).

**Table 5:** Associations between receiving treatment for child diarrhoea at private facilities and household sociodemographic characteristics

	2006 (***N*** = 315)	2011 (***N*** = 441)	2016 (***N*** = 240)
**Household characteristics**	**AOR (95% CI)**	**AOR (95% CI)**	**AOR (95% CI)**
**Wealth quintile**			
Poorest (*reference*)			
Second poorest	2.37 (0.91–6.20)	0.79 (0.35–1.82)	4.95* (1.44–16.98)
Middle	3.06* (1.19–7.90)	0.93 (0.46–1.87)	4.19* (1.25–14.04)
Second richest	3.65** (1.40–9.55)	1.95 (0.78–4.85)	3.95* (1.22–12.77)
Richest	8.03** (2.43–26.54)	3.34 (0.95–11.67)	6.41** (1.59–25.85)
**Caste/ethnicity**			
Dalit (*reference*)			
Janajati	0.93 (0.34–2.54)	0.80 (0.36–1.79)	0.53 (0.20–1.42)
Brahmin/chhetri	0.51 (0.19–1.37)	0.61 (0.27–1.34)	0.32 (0.11–0.90)
Other	1.17 (0.24–5.65)	0.44 (0.15–1.30)	0.50 (0.14–1.77)
**Mother’s years of schooling**	0.98 (0.89–1.09)	1.00 (0.91–1.11)	1.00 (0.90–1.11)
**Mother’s age**	1.00 (0.94–1.06)	0.95 (0.90–1.01)	1.03 (0.96–1.11)
**Female household head**	1.93 (0.88–4.25)	0.98 (0.53–1.80)	0.60 (0.28–1.28)
**Rural residence**	0.91 (0.38–2.17)	0.61 (0.27–1.35)	0.68 (0.29–1.56)
**Agroecological zone**			
Mountain (*reference*)			
Hill	0.87 (0.35–2.20)	0.66 (0.30–1.47)	2.31 (0.58–9.20)
Terai	1.41 (0.59–3.38)	2.47* (1.03–5.89)	4.17 (0.90–19.34)

* *p* < 0.05; ** *p* < 0.01

Across all three survey waves, children from the terai agroecological region were more than three times as likely as children from the mountain region to receive treatment for fever/cough at private facilities. In 2011 and 2016, children from the richest households were more than three times as likely as children from the poorest households to receive treatment for fever/cough at private facilities (Table 6).

**Table 6:** Associations between receiving treatment for child fever/cough at private facilities and household sociodemographic characteristics

	2006 (***N*** = 624)	2011 (***N*** = 903)	2016 (***N*** = 1193)
**Household characteristics**	**AOR (95% CI)**	**AOR (95% CI)**	**AOR (95% CI)**
**Wealth quintile**			
Poorest (*reference*)			
Second poorest	1.13 (0.48–2.70)	1.84 (0.96–3.52)	2.11** (1.22–3.65)
Middle	1.29 (0.58–2.84)	1.69 (0.81–3.54)	1.82* (1.00–3.34)
Second richest	1.32 (0.56–3.11)	2.89** (1.32–6.31)	1.90* (1.01–3.57)
Richest	2.45 (0.97–6.19)	3.48** (1.35–9.00)	3.68** (1.43–9.51)
**Caste/ethnicity**			
Dalit (*reference*)			
Janajati	1.46 (0.70–3.06)	1.31 (0.67–2.57)	1.04 (0.56–1.92)
Brahmin/Chhetri	1.38 (0.72–2.64)	1.37 (0.72–2.60)	0.59 (0.32–1.08)
Other	1.17 (0.39–3.50)	1.37 (0.61–3.09)	1.26 (0.56–2.82)
**Mother’s years of schooling**	0.95 (0.88–1.02)	0.96 (0.89–1.03)	0.97 (0.92–1.03)
**Mother’s age**	0.98 (0.94–1.01)	1.00 (0.96–1.04)	0.97 (0.94–1.01)
**Female household head**	1.02 (0.56–1.86)	1.30 (0.84–2.03)	0.75 (0.52–1.09)
**Rural residence**	0.63 (0.34–1.16)	0.98 (0.52–1.84)	1.14 (0.72–1.80)
**Agroecological zone**			
Mountain (*reference*)			
Hill	3.11** (1.32–17.30)	2.06* (1.02–4.16)	1.10 (0.50–2.42)
Terai	5.29** (2.32–12.10)	3.71** (1.93–7.14)	3.19** (1.37–7.47)

* *p* < 0.05; ** *p* < 0.01

## Discussion

This paper outlines trends in and sociodemographic characteristics associated with maternal and child health-seeking practices in Nepal using nationally representative survey data. The proportion of women receiving ANC services, institutional delivery, and treatment for child diarrhoea and fever/cough at private health facilities increased over time, with the highest proportion of women receiving health services in the private sector in 2016. Household wealth status, total years of schooling, and agroecological zone had the strongest associations with utilisation of maternal and child health services at private health facilities.

The present findings indicate that maternal and child health-seeking practices in the private sector increased from 2006 to 2016. A study based on 205 demographic and health surveys conducted in 70 LMICs between 1990 and 2013 also indicated an increase in health-seeking for maternal and child health services at private facilities over time [32]. Other studies indicate that in many LMICs, most people receive child health care services at private facilities, including private clinics and local pharmacies [33, 34]. In Nepal, private clinics and local pharmacies are the primary point of access for health services [35]. Furthermore, the number of private clinics and local pharmacies available has increased over the last two decades [36].

The increase in the use of private health services over time may also be due to increased attention to the public private partnership approach in health care services in both high-income countries and LMICs [37]. In Nepal, the 1991 National Health Policy provided avenues for private institutions to enter the health sector. The government in Nepal has emphasized the public private partnership approach in health care services. This has facilitated the expansion of private health institutions in both Kathmandu, the capital city, and other major cities [38, 39].

Household wealth status, mother’s years of schooling, and agroecological zone were key determinants for utilisation of private health services for maternal and child health care. A randomized controlled trial conducted among disadvantaged communities in Bangladesh, India, and Nepal from 2005 to 2011 indicated that institutional delivery was strongly associated with household wealth status and the mother’s level of education [40]. Similarly, a study based on nationally-representative survey data from 16 countries in sub-Saharan African, Latin America, and Asia indicated that delivery in the private sector significantly increased from 1997 to 2003. Household socioeconomic status was the key determinant associated with delivery at private facilities [41]. A trend analysis of the NDHS 2006 data showed economic disparities in access and utilisation of ANC and delivery services [42]. Selection of private health facilities was dependent on the economic status of the patient. Similarly, findings from a nationally representative cross-sectional survey in Bangladesh showed that household wealth determined health-seeking from private health facilities [43].

The increase in the use of private health services may partially be linked to increased purchasing power. An analysis conducted by the World Health Organization in 39 countries found that countries are increasingly relying on private services for outpatient care. Individuals from the highest wealth quintile are more likely to use private inpatient services than individuals from the poorest wealth quintile, while individuals from poorer households are more likely to rely on government facilities for inpatient services [44]. A recent study from India also indicated that wealth quintile is a major predictor for choice of health facility [45].

The quality of health services is a primary determinant for choice of health facility. Studies conducted in India, Pakistan, South Africa, Nigeria, Malawi, and Saudi Arabia have indicated that poor quality of care in public health services was a key determinant for shifting to private facilities [45–50]. There is widespread debate regarding the quality of services in private versus public facilities [33]. In Nepal, many individuals harbor concerns with the quality and effectiveness of health services in public facilities. These include concerns around availability of medicine and equipment, quality of health workers, and accessibility of service hours [51]. These concerns, and a general lack of confidence in public health facilities, could also account for the shift toward utilising private facilities for maternal and child health services in Nepal. Furthermore, women from the highest wealth quintile have the financial resources to adjust health-seeking behaviours based on such preferences and concerns.

Our study shows that, compared to other maternal and child health services, the highest percentage of women use public health facilities for delivery. This could be because of the government’s allowance system for institutional delivery through government health facilities introduced in 2005 [52]. A study conducted in Nepal in 2018 suggests that the institutional delivery rate has in fact increased with the implementation of the maternity incentive scheme program [52]. There are opportunities for similar government incentive schemes, for example, incentive programs for post-delivery home visits by midwives to provide PNC services. However, challenges with the maternity incentive scheme need to be addressed before scaling up incentive programs to other areas of maternal and child healthcare. Specific challenges include delays in receiving the incentive, the requirement that women complete an ANC card to receive the incentive, and lack of autonomy in choice of provider [53].

In recent years, the Nepal government has laid a strong foundation to improve maternal health services. For instance, the Interim Constitution in 2007 included basic health services and reproductive health as citizen rights. This was accompanied by a significant increase in the health budget, with a three-fold increase seen between 2006 and 2011. Likewise, the National Safe Motherhood and Newborn Health long term plan 2002–2017 was developed to strengthen the national health system, and the National Policy on Skilled Birth Attendant 2006 was endorsed by the government to acknowledge geographical and cultural barriers to accessing safe delivery services among the poorest and rural households [54]. These efforts have all increased access to maternal health services, even in hard-to-reach areas, and thereby reduced maternal mortality.

These findings should be interpreted with the following strengths and limitations in mind. Since we used nationally-representative data for the analyses, findings are generalizable to women across Nepal. The use of data from three survey waves further strengthens the present findings. Despite these strengths, there are existing limitations. First, child illness data only included the past 2 weeks; this could lead to selection bias. Second, a social health insurance scheme has been implemented in only some parts of Nepal which may have impacted women’s choice of health facility in these regions. Third, as this study analysed cross-sectional data, we were unable to determine causal relationships between sociodemographic characteristics and health-seeking behaviours.

The findings should also be interpreted with the public private partnership programs implemented in Nepal in mind, as these may have affected access, availability and quality of maternal and child health care services. For example, the Public Private Partnership (PPP) model in Nepal has been implemented through government, NGO, and community collaboration. Some of the public private partnership models in Nepal include family planning services through Family Planning Association of Nepal (FPAN); safe abortion practices through Meri Stopes; and the establishment of community hospitals in some districts and medical collages in larger cities [39, 53]. Affordable health services offered at health facilities through the PPP model, as well as availability and geographic accessibility, may have motivated some women to visit private health facilities for care.

Finally, while we included several potential confounders based on our prior knowledge of the Nepal context, other confounders may have impacted trends in and determinants of private health facility use. For example, we did not collect data on health insurance, or consider different confounders for maternal vs. child health-seeking. Furthermore, we did not collect data on service quality. Service quality may be an important determinant in the selection of a particular health facility (public or private). Hence, future research should measure service quality to assess its role in determining health-seeking behaviours for maternal and child health services.

## Conclusions

Increased utilisation of private facilities for maternal and child health care indicates the importance of a system that incorporates private health facilities alongside government facilities. These findings highlight important policy and practice implications. As more people utilise private health facilities, there is a need to institute monitoring and supervision mechanisms to ensure standards for quality health care are upheld, including mechanisms for timely and correct reporting to the national information management system. Currently, consultation and hospital fees are subjective and differ across private facilities; there is a strong need to systematize these expenses. Increased collaboration between public and private health facilities through public private partnerships would provide opportunities to guarantee the constitutional right to healthcare for all citizens. Additionally, the government needs to improve health services across facility levels to meet public requirements, while also strengthening other building blocks of the health system. While the government still struggles to cater to the health needs of people in hard-to-reach areas, maximizing availability of private facilities through increased collaboration with the private sector would guarantee the constitutional right to health care for all citizens. Engagement of the private sector through the PPP model can further improve capacity of health service providers, increase innovation, and strengthen collaboration, with the ultimate aim of strengthening health services, including maternal and child healthcare, in resource-limited setting.

## Supplementary Information

**Additional file 1: Supplementary Table 1**. Percent of women seeking ANC services at private facilities for each sociodemographic characteristic. **Supplementary Table 2**. Percent of women seeking delivery services at private facilities for each sociodemographic characteristic. **Supplementary Table 3**. Percent of women seeking child diarrhoea treatment for their children at private facilities for each sociodemographic characteristic. **Supplementary Table 4**. Percent of women seeking child fever/cough treatment for their children for each sociodemographic characteristic

## Data Availability

The datasets used in this study are available upon request from the corresponding author.

## Abbreviations

ANC: Antenatal Care
AOR: Adjusted Odds Ratio
CI: Confidence Interval
DHS: Demographic and Health Survey
LMICs: Low- and Middle- Income Countries
NDHS: Nepal Demographic Health Survey
PNC: Postnatal Care
PPP: Public Private Partnership
PPS: Probability Proportion-to-Size
