# Supplementary material for: Trends in and determinants of visiting private health facilities for maternal and child health care in Nepal: comparison of three Nepal demographic health surveys, 2006, 2011, and 2016
Source: BMC Pregnancy Childbirth. 2021 Jan 3;21:1. doi: 10.1186/s12884-020-03485-8 (PMC7778799; doi:10.1186/s12884-020-03485-8)
Supplement: Supplementary file 1 — Additional file 1: Supplementary Table 1. Percent of women seeking ANC services at private facilities for each sociodemographic characteristic. Supplementary Table 2. Percent of women seeking delivery services at private facilities for each sociodemographic characteristic. Supplementary Table 3. Percent of women seeking child diarrhoea treatment for their children at private facilities for each sociodemographic characteristic. Supplementary Table 4. Percent of women seeking child fever/cough treatment for their children for each sociodemographic characteristic [file 12884_2020_3485_MOESM1_ESM.docx]

**Supplementary Table 1: Percent of women seeking ANC services at private facilities for each sociodemographic characteristic**

|  | **2006** | | **2011** | | **2016** | |
| --- | --- | --- | --- | --- | --- | --- |
| **Household characteristics** | **(%)** | **N** | **%** | **N** | **%** | **N** |
| **Wealth quintile** |  |  |  |  |  |  |
| Poorest | 8.6 | 483 | 11.0 | 657 | 6.7 | 712 |
| Second poorest | 10.7 | 615 | 14.8 | 733 | 18.1 | 782 |
| Middle | 14.3 | 625 | 24.0 | 792 | 26.8 | 836 |
| Second richest | 24.3 | 642 | 38.9 | 701 | 31.9 | 801 |
| Richest | 38.6 | 635 | 55.4 | 637 | 44.9 | 631 |
| **Caste/ethnicity** |  |  |  |  |  |  |
| Dalit | 16.1 | 412 | 19.9 | 567 | 13.3 | 510 |
| Janajati | 19.0 | 1,040 | 24.1 | 1,220 | 21.7 | 1,218 |
| Brahmin/Chhetri | 24.7 | 1,043 | 34.4 | 1,154 | 26.2 | 1,111 |
| Other | 15.2 | 505 | 33.3 | 579 | 35.7 | 923 |
| **Mother’s years of schooling** |  |  |  |  |  |  |
| No schooling | 10.4 | 1,543 | 19.7 | 1,408 | 20.4 | 1,143 |
| 1-5 years schooling | 20.7 | 563 | 22.2 | 679 | 19.5 | 689 |
| 6-9 years schooling | 28.3 | 614 | 31.1 | 814 | 24.4 | 985 |
| 10 and above years of schooling | 52.4 | 280 | 50.8 | 619 | 36.3 | 945 |
| **Mother’s age** |  |  |  |  |  |  |
| Less than 20 years | 17.3 | 278 | 22.9 | 306 | 23.3 | 326 |
| 20-24 years | 25.0 | 1,085 | 28.5 | 1,179 | 25.4 | 1,227 |
| 25-29 years | 19.1 | 933 | 30.4 | 1169 | 27.6 | 1303 |
| 30 and above years | 14.2 | 704 | 27.1 | 866 | 22.6 | 906 |
| **Household head** |  |  |  |  |  |  |
| Male | 19.5 | 2,393 | 28.0 | 2,566 | 26.3 | 2,629 |
| Female | 21.7 | 607 | 29.2 | 954 | 23.1 | 1,133 |
| **Place of residence** |  |  |  |  |  |  |
| Urban | 32.8 | 471 | 43.4 | 392 | 28.3 | 2,122 |
| Rural | 17.5 | 2,529 | 26.4 | 3,128 | 21.5 | 1,640 |
| **Agroecological zone** |  |  |  |  |  |  |
| Mountain | 9.9 | 191 | 10.4 | 237 | 4.8 | 242 |
| Hill | 21.6 | 1,159 | 22.5 | 1,328 | 20.9 | 1,493 |
| Terai | 19.9 | 1,650 | 34.4 | 1,955 | 31.0 | 2,027 |
| Total | 19.9 | 3,000 | 28.3 | 3,520 | 25.3 | 3,762 |

**Supplementary Table 2: Percent of women seeking delivery services at private facilities for each sociodemographic characteristic**

|  | **2006** | | **2011** | | **2016** | |
| --- | --- | --- | --- | --- | --- | --- |
| **Household characteristics** | **%** | **N** | **%** | **N** | **%** | **N** |
| **Wealth quintile** |  |  |  |  |  |  |
| Poorest | 1.3 | 1,412 | 1.9 | 1,390 | 1.6 | 1,082 |
| Second poorest | 2.4 | 1,180 | 3.6 | 1,182 | 5.6 | 1,072 |
| Middle | 2.6 | 1,132 | 7.0 | 1,133 | 12.9 | 1,122 |
| Second richest | 6.5 | 983 | 15.5 | 938 | 15.9 | 1,036 |
| Richest | 13.8 | 838 | 28.4 | 748 | 21.7 | 748 |
| **Caste/ethnicity** |  |  |  |  |  |  |
| Dalit | 1.9 | 871 | 4.7 | 959 | 4.5 | 695 |
| Janajati | 3.9 | 2,005 | 8.5 | 1,892 | 11.8 | 1,590 |
| Brahmin/Chhetri | 7.2 | 1,725 | 13.0 | 1,618 | 12.3 | 1,396 |
| Other | 4.0 | 944 | 9.8 | 922 | 11.5 | 1,379 |
| **Mother’s years of schooling** |  |  |  |  |  |  |
| No schooling | 1.5 | 3,420 | 4.3 | 2,619 | 5.8 | 1,793 |
| 1-5 years schooling | 4.6 | 932 | 7.8 | 1,010 | 7.4 | 959 |
| 6-9 years schooling | 10.5 | 848 | 13.0 | 1,039 | 13.5 | 1,226 |
| 10 and above years of schooling | 21.3 | 345 | 24.8 | 723 | 19.3 | 1,082 |
| **Mother’s age** |  |  |  |  |  |  |
| Less than 20 years | 5.5 | 368 | 7.7 | 381 | 9.9 | 391 |
| 20-24 years | 5.5 | 1,931 | 9.5 | 1802 | 11.1 | 1665 |
| 25-29 years | 4.7 | 1,742 | 11.0 | 1736 | 10.2 | 1780 |
| 30 and above years | 3.1 | 1,504 | 7.7 | 1473 | 11.8 | 1224 |
| **Household head** |  |  |  |  |  |  |
| Male | 4.1 | 4,435 | 9.2 | 4030 | 11.5 | 3602 |
| Female | 6.8 | 1,110 | 9.9 | 1361 | 9.2 | 1458 |
| **Place of residence** |  |  |  |  |  |  |
| Urban | 9.1 | 677 | 19.6 | 503 | 12.5 | 2730 |
| Rural | 3.4 | 4,868 | 8.3 | 4888 | 8.9 | 2330 |
| **Agroecological zone** |  |  |  |  |  |  |
| Mountain | 1.7 | 483 | 2.6 | 428 | 1.7 | 361 |
| Hill | 3.9 | 2,261 | 5.7 | 2,130 | 9.9 | 1,911 |
| Terai | 5.7 | 2,802 | 13.2 | 2,833 | 12.7 | 2,788 |
| Total | 4.6 | 5,545 | 9.4 | 5,391 | 10.8 | 5,060 |

**Supplementary Table 3: Percent of women seeking child diarrhoea treatment for their children at private facilities for each sociodemographic characteristic**

|  | **2006** | | **2011** | | **2016** | |
| --- | --- | --- | --- | --- | --- | --- |
| **Household characteristics** | **%** | **N** | **%** | **N** | **%** | **N** |
| **Wealth quintile** |  |  |  |  |  |  |
| Poorest | 35.7 | 53 | 48.5 | 86 | 37.1 | 35 |
| Second poorest | 64.8 | 71 | 53.6 | 98 | 80.9 | 50 |
| Middle | 67.3 | 68 | 59.1 | 115 | 80.3 | 68 |
| Second richest | 68.4 | 65 | 74.7 | 89 | 77.4 | 55 |
| Richest | 82.9 | 58 | 81.9 | 53 | 82.0 | 32 |
| **Caste/ethnicity** |  |  |  |  |  |  |
| Dalit | 63.3 | 35 | 68.8 | 102 | 74.2 | 38 |
| Janajati | 70.0 | 114 | 71.6 | 119 | 75.8 | 58 |
| Brahmin/Chhetri | 51.4 | 107 | 55.6 | 120 | 54.6 | 45 |
| Other | 78.4 | 59 | 63.7 | 100 | 81.1 | 99 |
| **Mother’s years of schooling** |  |  |  |  |  |  |
| No schooling | 65.7 | 159 | 57.2 | 210 | 76.5 | 88 |
| 1-5 years schooling | 51.3 | 57 | 68.7 | 84 | 70.5 | 57 |
| 6-9 years schooling | 69.5 | 82 | 62.4 | 103 | 67.9 | 52 |
| 10 and above years of schooling | 73.6 | 17 | 68.4 | 44 | 79.5 | 43 |
| **Mother’s age** |  |  |  |  |  |  |
| Less than 20 years | 49.3 | 32 | 76.9 | 46 | 83.8 | 19 |
| 20-24 years | 67.2 | 119 | 63.1 | 163 | 70.4 | 86 |
| 25-29 years | 68.1 | 104 | 62.1 | 134 | 71.9 | 90 |
| 30 and above years | 61.3 | 60 | 51.8 | 98 | 79.6 | 45 |
| **Household head** |  |  |  |  |  |  |
| Male | 63.3 | 272 | 60.9 | 348 | 75.6 | 178 |
| Female | 72.2 | 43 | 65.0 | 93 | 68.5 | 62 |
| **Place of residence** |  |  |  |  |  |  |
| Urban | 77.6 | 40 | 78.7 | 44 | 78.8 | 125 |
| Rural | 62.6 | 275 | 59.8 | 397 | 68.4 | 115 |
| **Agroecological zone** |  |  |  |  |  |  |
| Mountain | 42.0 | 29 | 49.1 | 31 | 24.0 | 12 |
| Hill | 57.7 | 104 | 45.6 | 141 | 62.1 | 55 |
| Terai | 72.1 | 182 | 71.6 | 269 | 81.1 | 173 |
| Total | 64.6 | 315 | 61.7 | 441 | 73.8 | 240 |

**Supplementary Table 4: Percent of women seeking child fever/cough treatment for their children for each sociodemographic characteristic**

|  | **2006** | | **2011** | | **2016** | |
| --- | --- | --- | --- | --- | --- | --- |
| **Household characteristics** | **%** | **N** | **%** | **N** | **%** | **N** |
| **Wealth quintile** |  |  |  |  |  |  |
| Poorest | 53.6 | 99 | 55.5 | 100 | 59.1 | 150 |
| Second poorest | 62.1 | 112 | 72.2 | 187 | 83.1 | 246 |
| Middle | 65.1 | 124 | 72.2 | 210 | 86.7 | 314 |
| Second richest | 65.9 | 150 | 81.6 | 252 | 84.0 | 258 |
| Richest | 78.6 | 139 | 81.4 | 154 | 86.4 | 225 |
| **Caste/ethnicity** |  |  |  |  |  |  |
| Dalit | 60.3 | 89 | 66.4 | 135 | 80.6 | 139 |
| Janajati | 68.3 | 184 | 73.8 | 290 | 81.5 | 374 |
| Brahmin/Chhetri | 64.0 | 234 | 74.0 | 281 | 69.8 | 305 |
| Other | 70.8 | 117 | 82.0 | 197 | 92.5 | 375 |
| **Mother’s years of schooling** |  |  |  |  |  |  |
| No schooling | 66.5 | 300 | 76.7 | 347 | 86.0 | 378 |
| 1-5 years schooling | 63.9 | 111 | 70.1 | 155 | 79.5 | 205 |
| 6-9 years schooling | 65.4 | 152 | 72.4 | 239 | 80.9 | 301 |
| 10 and above years of schooling | 68.1 | 61 | 77.4 | 162 | 79.4 | 308 |
| **Mother’s age** |  |  |  |  |  |  |
| Less than 20 years | 70.2 | 62 | 74.4 | 82 | 84.4 | 102 |
| 20-24 years | 69.9 | 249 | 75.7 | 359 | 84.7 | 411 |
| 25-29 years | 65.0 | 177 | 71.3 | 283 | 81.9 | 432 |
| 30 and above years | 57.9 | 136 | 77.4 | 179 | 76.2 | 248 |
| **Household head** |  |  |  |  |  |  |
| Male | 66.1 | 492 | 73.5 | 652 | 83.9 | 845 |
| Female | 65.4 | 132 | 77.2 | 251 | 77.0 | 348 |
| **Place of residence** |  |  |  |  |  |  |
| Urban | 79.0 | 119 | 77.7 | 113 | 81.7 | 723 |
| Rural | 62.9 | 505 | 74.1 | 790 | 82.1 | 470 |
| **Agroecological zone** |  |  |  |  |  |  |
| Mountain | 30.8 | 33 | 44.0 | 38 | 60.2 | 46 |
| Hill | 62.1 | 263 | 65.7 | 269 | 68.7 | 401 |
| Terai | 72.5 | 328 | 80.5 | 596 | 90.3 | 746 |
| Total | 66.0 | 624 | 74.5 | 903 | 81.7 | 1193 |
